# p53-Induced Growth Arrest Is Regulated by the Mitochondrial *SirT3* Deacetylase

**DOI:** 10.1371/journal.pone.0010486

**Published:** 2010-05-05

**Authors:** SiDe Li, Michaela Banck, Shiraz Mujtaba, Ming-Ming Zhou, Mary M. Sugrue, Martin J. Walsh

**Affiliations:** 1 Department of Pediatrics, Mount Sinai School of Medicine, New York, New York, United States of America; 2 Department of Structural and Chemical Biology, Mount Sinai School of Medicine, New York, New York, United States of America; 3 Department of Medical Oncology, Mayo Clinic, Rochester, Minnesota, United States of America; Roswell Park Cancer Institute, United States of America

## Abstract

A hallmark of p53 function is to regulate a transcriptional program in response to extracellular and intracellular stress that directs cell cycle arrest, apoptosis, and cellular senescence. Independent of the role of p53 in the nucleus, some of the anti-proliferative functions of p53 reside within the mitochondria [1]. p53 can arrest cell growth in response to mitochondrial p53 in an EJ bladder carcinoma cell environment that is naïve of p53 function until induced to express p53 [2]. TP53 can independently partition with endogenous nuclear and mitochondrial proteins consistent with the ability of p53 to enact senescence. In order to address the role of p53 in navigating cellular senescence through the mitochondria, we identified SirT3 to rescue EJ/p53 cells from induced p53-mediated growth arrest. Human SirT3 function appears coupled with p53 early during the initiation of p53 expression in the mitochondria by biochemical and cellular localization analysis. Our evidence suggests that SirT3 partially abrogates p53 activity to enact growth arrest and senescence. Additionally, we identified the chaperone protein BAG-2 in averting SirT3 targeting of p53 -mediated senescence. These studies identify a complex relationship between p53, SirT3, and chaperoning factor BAG-2 that may link the salvaging and quality assurance of the p53 protein for control of cellular fate independent of transcriptional activity.

## Introduction

Mechanisms for nuclear p53 that mediate cellular senescence are largely known, however, the biochemical role of p53 in other organelle systems is less understood. Furthermore, the potential of so –called “p53 bodies” that exist within distinct cellular compartments to communicate between one another and with the cellular infrastructure has not been carefully examined. Until recently, the prevailing model for p53 function has been studied primarily within the nucleus with the exception of studying the stability of the p53 protein itself. A number of studies now confirm that p53 can mediate a program of cellular fate independent of a nuclear and transcriptionally- competent species of p53 through the association of the permeable potential of the inner mitochondrial membrane that confers the release of pro -apoptotic caspases [3]. Recent studies indicate that p53-induced senescence within nuclear PML bodies is directly antagonized by the protein deacetylase SirT1, a yeast *Sir2* homologue found in mammals, through the inactivation of p53 by deacetylation [4]. However, this contention has been somewhat challenged on the basis that the loss of SirT1 activity on p53 acetylation by a specific SirT1 inhibitor is likely compensated for by several others HDACs or sirtuins independent of SirT1 and thus fails to clearly demonstrate the unifying role for SirT1 on p53 -induced senescence [5]. Alternative rationale may place p53 elsewhere from SirT1 activity in a cellular context to mediate cellular senescence.

Human sirtuins are NAD+-dependent protein deacetylases expressed as a multigene family of seven distinct gene products (SirT 1-7) widely expressed in different tissues and localized within different subcellular compartments [6]. [7] [8]SirT1 directly mediate transcriptional processes involving gene silencing [9] and may regulate telomere function [10]. Additional findings suggest that murine sirtuin 1 (SirT1) directly mediate deacetylation of p53 that disrupt normal developmental processes [11]. In addition to SirT1, SirT3 is a member of the class I sirtuins, the most conserved members of the *Sir2* gene from *S. Cerevisiae*. SirT3, which is primarily localized within mitochondria and widely expressed in adult and fetal tissue, contains protein deacetylase activity [12], [13], [14] and expresses the major proportion of mitochondrial protein deacetylase activity [12]. A function of SirT3, like SirT1 and 2, is to deacetylate histones specifically at H4K16 where SirT3 translocation to mitochrondria was identified only during cellular stress [15] [16]. Furthermore, SirT3 protects cardiomyocytes from stress –induced cell death by deacetylating Ku70 and this promotes interaction of Ku70 with the proapoptotic protein Bax. Thus, under stress conditions, increased expression of SirT3 protects cardiomyocytes, in part by hindering the translocation of Bax to mitochondria [17] and is linked to the blocking of the cardiac hypertrophic response [18]. Surprisingly, SirT3 –deficient mice fail to display any profound phenotypic alterations in metabolism, cell growth, or adaptive thermogenesis, even when metabolically challenged, suggesting the possible redundancy in mitochondrial function by other sirtuins [19] [20], [21].

The need for the cellular translocation of p53 is in part guided through the chaperoning and the tight quality control of p53 for the proper conformation throughout cell compartments [22]. To execute the proper folding and shuttling of p53 throughout multiple cell compartments p53 relies on co-chaperoning factors such as Hsp90 [23] and is independently associated with the carboxyl terminus of Hsp70-interacting protein (or CHIP) to direct proteosomal sorting of mutant p53 [24] and to salvage wild-type or misfolded p53 from degradation [25]. However, the ubiquitin ligase activity of CHIP on p53 is likely under tight regulation but the mechanics behind this control remain unclear. It is suspected that under various cellular and physiological conditions, and within different cellular compartments, there are differential and specialized roles of co-chaperones of p53 to accommodate the need for highly -ordered p53 conformation. As a result p53 is likely guided through several interactions with chaperones molecules with some of those needed to salvage or restore normal properties from mis-folded or mutant p53 species in different cell compartments. As a component of the CHIP containing complex, the BCL2 anthagene protein, BAG-2, was identified to inhibit CHIP to control for the destructive activity of the CHIP ubiquitin ligase on specific cellular proteins *in vivo* [26]. This may implicate BAG-2 as an important mediator that maintains the pools of native p53 throughout the cell. Recently, it has been reported that the tumor suppressor protein Tid1 (mtHsp40) promotes p53 translocation to mitochondria under hypoxic conditions to reinforce apoptosis, by masking p53 from degradation ensuring the trafficking to mitochondria [27].

In an effort to further elucidate alternative mechanisms regulating p53-induced senescence, we previously found that p53-induced senescence in EJ-p53 cells is characterized by a dramatic decrease in mitochondrial membrane potential, which is not responsive to agents that activate or inhibit the mitochondrial permeability transition pore complex (PTPC), e.g. atractyloside (ATR) and cyclosporin A (CSA), respectively, as well as decreased expression of adenine nucleotide translocase (ANT), the major protein in the mitochondrial inner membrane [28]. Furthermore, increasing evidence suggests that mitochondrial isoforms of normal p53 induce mitochondrial permeablization leading to the release of apopotosis –activating caspases [29], whereas, some tumor derived mutation fail to do this [30]. Interestingly, these processes likely couple p53 to many mitochondrial functions that include the redox pathways and energy metabolism of the cells [14]. Despite the assumption that sirtuins benefit cell survival through enzymatic processes, the precise substrates for this action have yet to be determined. Although recent evidence suggest that post-translational modification of p53 is not a requirement for targeting p53 to the mitochrondria [31], not all post-translational modifications have been excluded due to the lack of reagents necessary to test for all post-translational modifications of p53. We indicate that a synthetic p53 peptide can serve as a substrate for SirT3 *in vitro*. In this study we identify the co -localization of p53 with both nuclear and mitochondrial markers. Additionally, we identify the sirtuin protein SirT3, as a regulator of p53 –induced senescence and a marker for cellular lifespan and survival. Finally, we demonstrate that p53 interacts with the BAG-2 component of the CHIP ubiquitin ligase complex through the domain specifying mitochondrial –associated senescence domain (MASD) that challenge the ability of SirT3 to avert senescence by p53. These studies reveal a novel network whereby sirtuins and co-chaperones of p53 may coordinate cellular senescence in response to the presence of p53.

## Results and Discussion

### p53 fractionates and localizes with mitochondrial proteins during p53 -induced senescence

**Figure 1:**
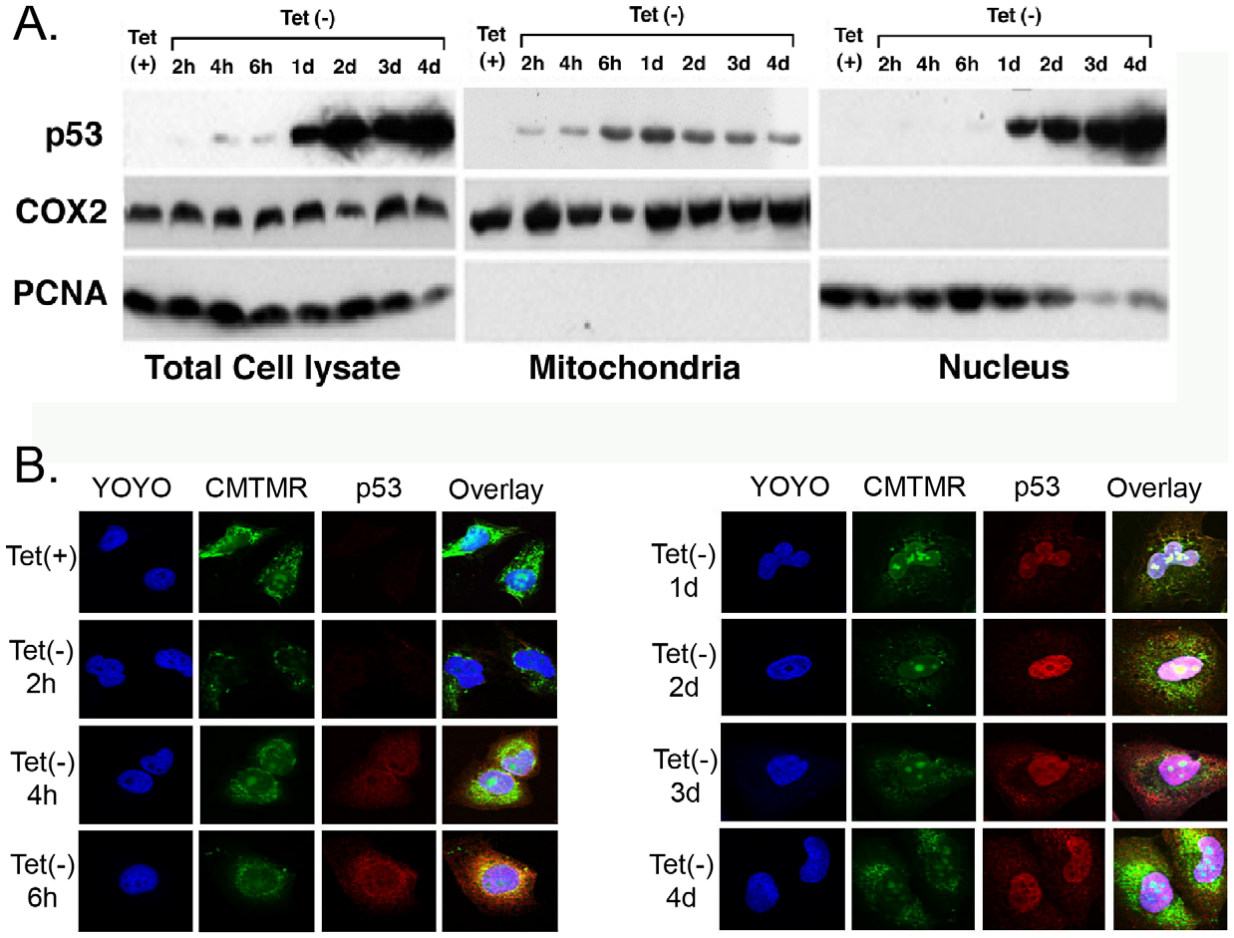
Analysis of p53 in subcellular fractions from senescent EJ-p53 cells. (**A**) Immunoblots using 50 µg protein of total cell lysate and subcellular fractions (mitochondria and nuclear fraction obtained from a sucrose gradient) were subject to SDS-PAGE and subsequently transferred to PVDF filters (Immobilon, Millipore Corp.). Blots were then blocked with BSA and initially probed using a mouse monoclonal antibody specific for p53 (clone 1801, Oncogene Research) followed by HRP-conjugated anti-mouse IgG (Roche) and then developed using ECL kit (GE Healthcare/Amersham). Blots were reprobed using mouse monoclonal specific for the mitochondrial marker, Cox II (Molecular Probes, Invitrogen) and nuclear marker PCNA (Calbiochem) followed by HRP-conjugated anti-mouse IgG and then developed using the ECL kit (GE Healthcare/Amersham). (**B**) Laser confocal image of p53 immunohistochemistry and CMTMR, YOYO fluorescence in EJ-p53 cells. EJ-p53 cells were maintained in (+) tet (no p53 expression) and (-)tet 2 hours through 4days (overexpression of wt p53). p53-specific mAb(Oncogene research) and Cy5-labled anti-mouse were used to immunohistochemistry localize p53 in EJ-p53 cells. YOYO was used to stain nuclei and CMTMR was to stain mitochondria. For each image, p53 was re-colored to red, Nuclei was re-colored to blue, and mitochondria was re-colored to green. p53 localization in p53-induced senescence in EJ-p53 cells was determined by overlay.

EJ-p53 cells were harvested at different time points as indicated (**Fig. 1A**) (+tet, -tet 2 hours, 4 hours, 6 hours,1 day, 2 days,3 days and 4 days). Using a subcellular fractionation and sucrose step gradient, total cell lysates, nuclear and mitochondrial fractions were obtained. Specific nuclear and mitochondrial subcellular organelle markers were used to trace p53 alongside the fractionation procedure. Upon tetracycline withdrawal p53 was detected in mitochondrial fractions as early as 4 h after and persisted in all subsequent time points analyzed, e.g. -tet 6 h, 1 d, 2 d, 3 d, 4 d. From –tet 1 day to 4 days, p53 levels dimished (**Fig. 1A**). However, p53 levels were higher in nuclear and total cell lysates prepared from EJ-p53 cells over the same time course. Immunohistochemical analysis using confocal imaging showed p53 exclusively in mitochondria at –tet 2 h (**Fig. 1B**). At –tet 4 h, p53 was detected at higher levels in the mitochondria with concomitant appearance of p53 in the nucleus. p53 persisted in mitochondria and the nucleus for the duration of the time course studied (from −4 h through −4 d).

### Structural requirements for p53 –induced senescence in EJ-p53 bladder cacinoma cells

**Figure 2:**
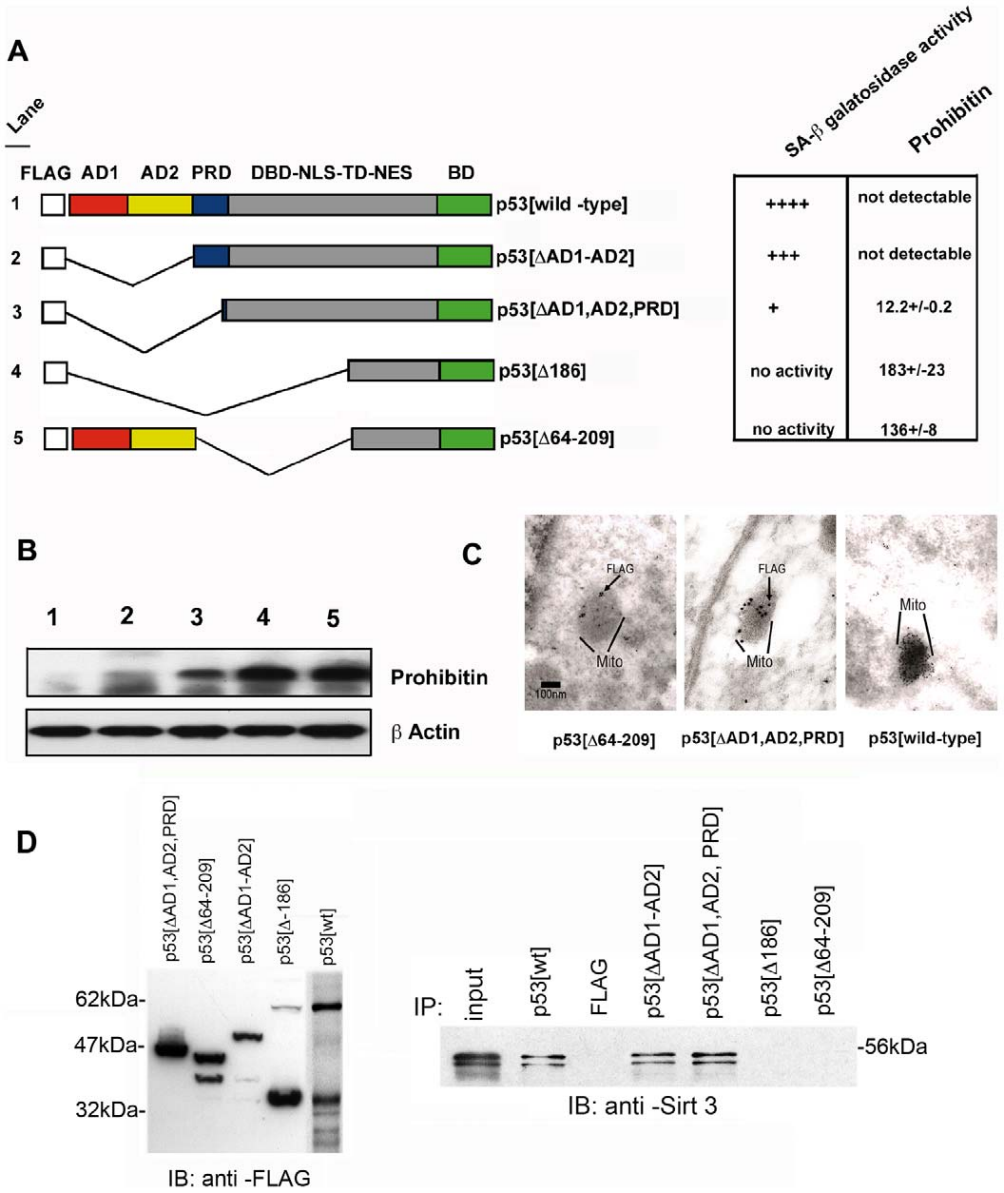
Deletion of p53 identifies the novel mitochrondria -associated senescence domain (MASD) between amino acids 64 and 209 of p53. (**A**) Various deletions of FLAG –tagged p53 were used to determine involvement in mitochrondria –associated senescence programs in EJ-p53 cells. The following abbreviations were used to characterize individual domains within p53 as, AD1, activation domain 1; AD2, activation domain 2; PRD, proline rich domain; DBD-NLS-TD-NES, combined DNA binding domain-nuclear localization signal-transactivation domain-nuclear export signal; BD, basic domain. Following the transient transfection of individual p53 constructs into the uninduced EJ-p53 cells, expression of p53 protein levels were normalized versus cell number to measure the level of SA -β galactosidase activity by staining with Xgal. ELISA analysis was then performed using antisera against human prohibitin (Research Diagnostics, Inc.). Colormetric analysis was then used to measure the amount of SA-β galactosidase activity ELISA was performed to measure prohibitin levels (fg/ml lysate) and normalized by the amount of immunoprecipitated FLAG-tagged p53 protein used as input from the ELISA assay. (**B**) Immunoblot analysis was then performed with anti –prohibitin nitrocellulose filter was reused to immunoblot with an anti- β actin polyclonal antisera (Sigma-Aldrich). (**C**) Electron micrograph (10,000X) of EJ carcinoma cells transfected with the different FLAG-tagged and truncated variants of human p53 and stained with the anti-FLAG monoclonal antibody (Sigma-Aldrich). Region corresponding to the outline of the mitochrondria is indicated. (**D**) Interaction of Sirt3 with the MASD region of p53. Using FLAG-tagged variants of the deleted p53 cDNAs expressed by transient transfections of p53 shown (left) were used to identify specific interactions with endogenous Sirt3 by immunoprecipitation with M2 agarose (Sigma-Aldrich) followed by standard immunoblotting protocols.

One of the profiles for p53 when overexpressed is growth suppression and arrest [32]. Using the expression of the wild –type form of p53, we have previously demonstrated the immediate response that follows to p53 induction in the EJ bladder carcinoma system is cellular growth arrest/premature arrest initiated by transfected p53 [2], [33]. We have mapped domains of p53 relevant for premature growth arrest in EJ bladder carcinoma cells expression by measuring levels of senescence –associated (SA) β galatosidase activity [34] and levels of prohibitin as having an counter-correlative relationship to growth arrest [35] by ELISA and immunoblot analysis (Research Diagnostics, Inc.) from EJ-p53 cell lysates. To confirm the ability of p53 to induce cell growth arrest we have transfected p53 plasmids into the EJ bladder carcinoma cell line [36]. Using truncations of p53 within the FLAG-tagged p53 cDNA plasmid constructs, designed to ablate specific domains as shown (**Fig. 2A**), we assessed levels of SA β galactosidase staining and prohibitin protein expression. Following 48 hours after transfection parallel experiments were performed using cell lysates recovered for ELISA analysis and immunoblotting for prohibitin (**Fig. 2A & B**). In performing these studies we have identified the proline –rich (PRD) and DNA binding (DBD) domains as important structural determinants for their relative contribution in promoting SA-β galatosidase activity and decreases in prohibitin levels. Results of these studies indicate a cumulative contribution of the PRD and DBD in mitochondria -associated senescence in the EJ bladder carcinoma cells (**Fig. 2A & B**). However, significant contribution was made in p53 –induced senescence when the DBD remained intact. Shown is the summary of experiments conducted to determine the correlation between different domains of p53 and the ability of the different domains of p53 to stimulate senescence –associated (SA) β galatosidase activity (**Fig. 2A**) are correlative with mitochondrial occupation from electromicrographs of transfected cells (**Fig. 2C**). Deletion of the PRD results in a substantial loss of growth arrest, however, deletion of the DBD domains results in a greater loss in cell growth arrest when compared to the full-length and wild type form of p53 using tetracycline inducible expression. However, absence of both PRD and DBD regions results in the complete loss of SA-β galactosidase activity and a dramatic increase in prohibitin protein levels (**Fig. 2B**). To identify p53 interactions with SirT3 we used FLAG –p53 constructs shown (Fig. 2A) to transiently transfect the EJ-p53 cell line, without the prior induction of p53, and determine the association with endogenous SirT3 from whole cell extracts. We identify that p53 requires the central domain encompassing residues from 69 to residue 204 as important for the interaction between p53 and SirT3 (Fig. 2D).

### Rescue of the growth arrest phenotype by inducible p53 involves the mitochondrial NAD –dependent Sir2 –like sirtuin protein, SirT3

**Figure 3:**
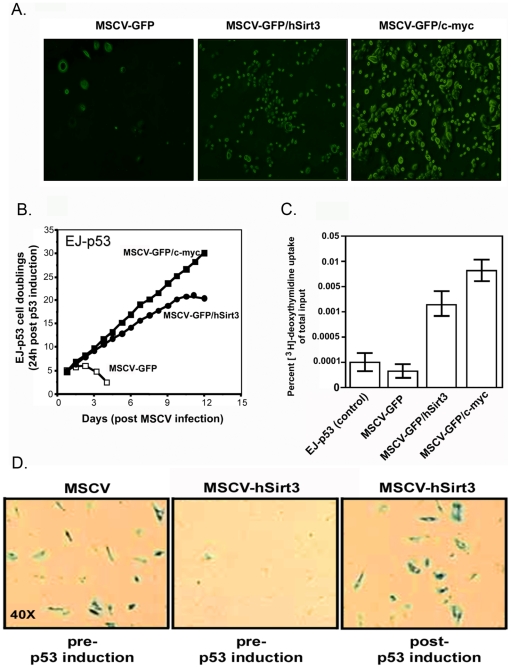
The NAD+ -dependent *Sir2* -like mitochondria protein deacetylase, SirT3, rescues EJ-p53 from p53 –inducible growth arrest. The human SirT3 2.9 kb cDNA identified in the retroviral screen (**Fig. 2**) was placed in the MSCV retroviral plasmid using a bicistronic cassette with GFP (Green fluorescent protein). EJ-p53 cells were transduced with MSCV particles containing only GFP, mouse *c-myc* and human SirT3. Cell growth kinetics were determined by laser scanning florescence microscopy (**A**) and by monitoring cell populations (**B**). (**C**) [^3^H]-thymidine incorporation studies were assayed after 72 hours in EJ-p53 cells following transduction with indicated retroviruses followed by induction of p53 expression by tetracycline withdrawal for 72 hours. (**D**) SA-β galactosidase activity were determined in retroviral transduced EJ-p53 with MSCV-GFP/Sirt3 transduced into EJ-p53 cells prior and following induction of p53 by tetracycline withdrawal.

Senescence can be a model system to identify genes that mitigate p53 function involved in the development of cancer, whereby, normal p53 function is compromised to promote cellular transformation and extends cellular lifespan. In our effort to demonstrate what specific factors can rescue the growth arrest phenotype generated by inducible p53 expression in the EJ bladder carcinoma cell system, we reasoned that the identification of gene products bypassing growth arrest could allow us to identify further regulatory mechanisms involved in averting the early -immediate p53 -induced senescence programs, a likely target for promoting oncogenesis [37]. With this aim, we performed a functional screen for cDNAs that could avert the growth arrest phenotype in the EJ –p53 inducible cell model. EJ –p53 cells prior to senescence (24 pre-induction) were infected with a retroviral MaRX cDNA library prepared from embryonic mouse fibroblasts [38]. MaRX retroviral vectors have a loXp site incorporated in the long-terminal repeat (LTR) and a miniplasmid between the LTRs for improved retroviral rescue. After 24 of mock infection, EJ –p53 cells were induced by withdrawal of tetracycline resulting in complete senescence of control cells. A senescence associated (SA) β-galactosidase staining was used to monitor infected EJ-p53 following p53 induction for senescence and growth arrest (**Fig. 3D**). Proliferating cells from the cDNA library -infected plates were monitored for [^3^H] thymidine incorporation were pooled and integrated proviruses were recovered by *in vitro Cre* recombinase excision, taking advantage of the loxP site incorporated in the MaRX LTR.[38] Sequencing of the recovered proviruses rendered several different cDNAs, but only infection with retroviral clone A478-34, which contains a 2.9 kb insert representing the full cDNA coding for a NAD+ dependent Sir2 –like protein, SirT3, rescued EJ-p53 cells completely from growth arrest.

There are seven mammalian *Sir2* homologs (SirT1-7) [39], all of which maintain the catalytic core domain homologous to yeast *Sir2*. NAD-dependent protein deacetylase activity has been demonstrated for mammalian SirT1, SirT2, and SirT3 proteins. The presence of NAD-dependent ADP-ribosylase and protein deacetylase activities of sirtuin proteins suggests that they may function as sensors of metabolic or oxidative states of cells and regulate cellular functions accordingly. Mammalian SirT1, which resides in the nucleus, is the most closely related to yeast *Sir2* and SirT3 is most closely related to SirT1 [8]. SirT1 binds and deacetylates p53 [40] NF-kB [41], and histones [42]. In contrast, mSirT1-deficient cells are p53-hyperacetylated and have elevated p53-dependent apoptosis, whereas, mSirT1 knock-out mice exhibit developmental defects [11], [14]. Human SirT3 is a mitochondria protein with its closest homolog being SirT1, with its N-terminal 25 amino acid residues partially responsible for its mitochondrial localization [12], [13]. Synthesized as an enzymatically inactive protein, human SirT3 is activated by a mitochondrial matrix-processing peptidase[12]. Compared with human SirT3, however, murine SirT3 lacks the N-terminal 142 amino acid residues necessary for the mitochondria localization for the human version. Nevertheless, the murine SirT3 was shown to maintain a paranuclear localization, consistent with a mitochondrial distribution pattern [12].

To determine further whether SirT3 has the capacity to avert growth arrest and to extend cell longevity we evaluated the role of SirT3 to increase the replicative potential of EJ-p53 cells. Shown in **Fig. 3** we infected EJ-p53 cells (5×10^5^ cells per 30 mm well/MOI 50 particles per cells) 24 hours prior to tetracycline withdrawal with three separate retroviruses shown carrying the bicistronic EGFP cassette lacking an insert (MSCV-GFP) and carrying either the full length human SirT3 (MSCV-GFP/hSirT3) or c-myc (MSCV-GFP/c-myc) cDNAs. After 24 hours of infection tetracycline was withdrawn from the medium for the induction of p53 and cell numbers were measured by fluorescence microscopy (**Figs. 3A and 3B**). After 72 hours, cell populations were monitored by fluorescence microscopy and indicate the capacity of both SirT3 and c-myc to avert the p53 –induced cell growth arrest in the EJ-p53 cells, whereas, the control virus containing the GFP cassette alone failed to engage cell proliferation. To accurately monitor the the EJ-p53 cell population following transduction by MSCV-GFP, MSCV-GFP/SirT3, and MSCV-GFP/c-myc and after 24 hours of induced p53 expression the EJ-p53 cell population was counted to determine the cell doublings. Results show that MSCV-GFP/*c-myc* can immortalize the EJ-p53 cell line after p53 induction, whereas, MSCV-GFP/SirT3 extended the doubling potential of the EJ-p53 cells for 10 days before succumbing to growth arrest (**Fig. 3B**). However, the MSCV-GFP control shows that cell doublings continue only over a short period of two days after induction of p53 expression. These data are consistent with the microscopy results (**Fig. 3A**) suggesting that SirT3 can avert p53 –induced growth arrest to extend the proliferative potential of the EJ-p53 cells. To demonstrate whether cell doublings are consistent with the DNA replicative potential of cells transduced with MSCV-GFP, MSCV-GFP/SirT3 and MSCV-GFP/*c-myc*, [^3^H]-thymidine incorporation studies were conducted. Results here indicate that [^3^H] thymidine incorporation is greatest with those cells transduced with GFP/*c-myc* after 24 hours of the induction of p53 expression (**Fig. 3C**). However, transduction of EJ-p53 with MSCV-GFP/Sirt3 was comparatively less than with GFP/*c-myc* but substantially greater than the control MSCV-GFP vector. Therefore, our results suggest that SirT3 expression can avert growth arrest by p53, however, the extension in cellular lifespan is temporary and can not immortalize the cells like MSCV-GFP/*c-myc* (**Fig. 3B**). Explanation of this may be due to the fact that upon p53 expression the temporary rescue from p53 growth arrest maybe confined to the mitochrondria localization. The nuclear localization of p53 may overcome the initial subcellular fraction of p53 in the mitochrondria to direct p53 senescence programs in the nucleus. SirT3 expression prior to p53 induction was required to completely avert growth arrest in the EJ-p53 cells (**Fig. 3D**). Therefore, the results indicate that commitment of cell fate is determined upon initial expression of p53 or SirT3. Hence, the expression of SirT3 is required prior to induction of p53 to avert the p53-mediated growth arrest phenotype (**Fig. 3D**).

### SirT3 associates with Bcl2 and p53 *in vivo*

**Figure 4:**
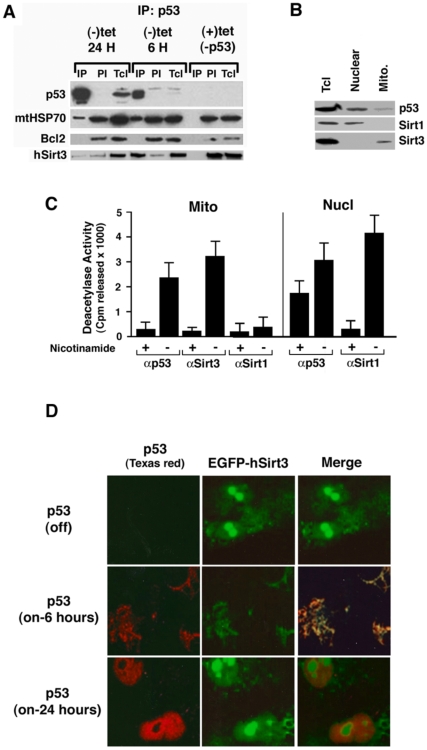
Association of p53 with mitochondrial proteins contains NAD+ dependent protein deacetylase activity of human SirT3. (**A**). Total cell lysate from (+)tet, (-)tet 6 h and 24 h EJ-p53 cells were collected and solubilized in lysis buffer containing complete proteolysis inhibitor. Monoclonal antibody against human p53 (DO1) and then protein G-agarose beads were added to the sample. The immunoprecipitated proteins from the washed beads were separated on a 10% gel, then transfer to filters for immunoblotting. Blots were blocked and probed with anti-p53 (mouse monoclonal DO-1), polyclonal rabbit anti-mthsp 70 (HSPA9) and mouse monoclonal anti-Bcl 2 (clone 124, Dako USA) and anti- SirT3 followed by HRP-conjugated anti-goat, HRP-conjugated anti-mouse and HRP-conjugated anti-rabbit, and then developed using ECL. (**B**). Total cell, nuclear, and mitochondrial fractions were obtained from EJ-p53 cells after 12 hours of tetracycline withdrawal and p53 expression. Each fractionated lysate was analyzed by SDS-PAGE and immunoblotted with antibodies against p53, SirT1 and SirT3. (**C**) Protein deacetylase activity was measured against a synthetic peptide corresponding to the human p53 protein sequence (HLKSKKGQSTSRHKKLMFK-C*) radiolabeled with [^14^C]-acetylCoA (GE Healthcare) and purified acetyltransferases CBP and PCAF *in vitro*. Deacetylase activity was determined from mitochondrial and nuclear fractions taken from EJ-p53 cells following 12 hours after induction of p53 expression by tetracycline withdrawal. Deacetylase activity was determined from p53, Sirt1, and Sirt3 immunopreciptates taken from nuclear and mitochondrial fractions. (**D**) Co-localization of p53 and SirT3 was performed by transient expression of human Sirt3 tagged with the Green Fluorescent Protein (GFP) and visualized by laser confocal microscopy. Inducible expression of p53 was monitored after 6 and 24 hours post-induction through the withdrawal of tetracycline (Tet) from growth medium. Separate images at each wavelength were merged to determine the signal overlay.

Identification of the retroviral clone A478-34 as the SirT3 cDNA reveals that mammalian SirT3 may have a direct role in regulating p53 –induced cell growth arrest in the EJ-p53 cell line. Furthermore our studies indicate that SirT3 can direct cell fate prior to the inducible cell growth arrest by p53, as shown (**Fig. 3**). New evidence reveals that p53 may be acetylated at the post-translational level prior to entering the nucleus. This finding has important implications for p53 –mediated senescence since acetylation of p53 at the intracellular level plays a critical role in navigating senescence by p53. As a result a large amount of effort was needed to create the necessary reagents to appropriately investigate this new area of p53 function. Specifically, it was necessary to produce new antisera for the post-translational modifications of p53 not commercially available. This includes antisera for the acetylated form of p53 unique to the mitochondrial form of p53 in the cell model used. Our study indicates that p53 is a unique target for the anti-senescence protein deacetylase called SirT3 (**Fig. 4**). SirT3, like the Sirt1 protein (previously known to deacetylate p53 in nucleus) can also deacetylate a synthetic peptide corresponding to p53 (**Fig. 4A**). However, this deacetylation by SirT3 seems to occur in the mitochrondria (**Fig. 4C**). New reagents for these novel investigations now require the generation of new expression vectors for both SirT3 and p53 as well as other targets for p53 function in the mitochrondria. These results indicate post-translational acetylation of p53 is significant for understanding the role of p53 in mitochrondria in promoting growth arrest.

Our results show the localization of p53 with the mitochondrial sirtuin protein, SirT3, is directly coupled to deacetylation of p53 in the mitochondrial compartment within the EJ-p53 cell line (**Fig. 4A–D**). Furthermore, we detect direct interactions between SirT3 and p53 through the MASD region of p53. (**Fig. 2D**). The lack of evidence from previous studies to support this claim [31] may have been due to the lack of high-quality antibody reagents to detect specific modifications of p53, which became available only after the previous studies. Although It is still unclear why antibodies directed at acetylated p53 failed to detect the acetylated species in the earlier report [31], one explanation may be conformational differences between nuclear and mitochondrial species of p53. However, recent evidence points to a role for the monoubiquitylation of p53 for trafficking to mitochrondria followed by deubiquitylation and stabilization of p53 with HAUSP [43] and may, therefore, indicate a transition to a monoubiquitated p53. This is an important finding given how little is known regarding p53 function in the mitochrondria even with important recent advances [43], [44]. There is even less known regarding the state of p53 acetylation in mitochrondria and the potential for signaling between modified species of p53. Previous studies have documented the interactions and deacetylation of p53 by Sirt1 [45], [46]. However, the biological implication of this are being challenged and suggest a more complicated mechanism for sirtuin protein regulation of p53 *in vivo* [5]. Our result now shows that p53 co-localizes with the protein deacetylase SirT3 in the mitochrondria prior to enacting irreversible growth arrest (**Fig. 4D**). Affinity purification and co-immunpreciptation studies of p53 and SirT3 illustrate the interactions between SirT3 and p53 in mitochondrial fractions (**Fig. 4A**). For confirmation of mitochondrial p53 interaction with mtHSP70 we show that the interaction between mtHSP70 and p53 is detectable (**Fig. 4A**). Comparatively, the relative absence of SirT3 in the nucleus indicates that this interaction may only take place in mitochrondria upon cellular stress [16], which is likely a consequence of elevated p53 expression in the EJ cells; whereas, Sirt1 co-fractionates with p53 in the nucleus (**Fig. 4B**). These results also confirm that the deacetylase activity expressed by SirT3 can deacetylate a specific p53 peptide sequences previously acetylated by histone acetyltransferases CBP and PCAF (**Fig. 4C**). Recent studies now indicate the functional capacity of cytosolic CBP/p300 to acetylate the type I interferon receptor to enforce signaling through the IFN enhancesome [47]. However, attempts to identify and define active p53 deacetylation in mitochrondria fail to identify specific acetylated species of p53 using combinations of polyclonal antibodies recognizing acetylated p53 for SirT3 -mediated deaceylation, yet SirT3 can deacetylate p53 *in vitro*. Recently, it has been suggested that SirT3 may distinguish between basal *versus* stress –induced apoptotic pathways evaluated in different cancer and epithelial cell lines and may be the result of maintaining control of the apoptotic pathway induced through the absence of Bcl2 [48]. However, the salvage of NAD+ levels in mitochrondria through the NAM phosphosribosyltransferase gene product (Nampt) requires intact SirT3 for maintaining an anti-apoptotic response to increase cellular survival [49]. In light of these discrepancies in the outcome of cellular survival from these studies further global proteomic analyses hope to distinguish pathways and proteins essential for mediating cell survival by SirT3.

### Interaction of p53 with BCL2 -associated athanogene (BAG-2) is mediated through the MASD of p53 to abrogate deacetylation of p53 by SirT3

**Figure 5:**
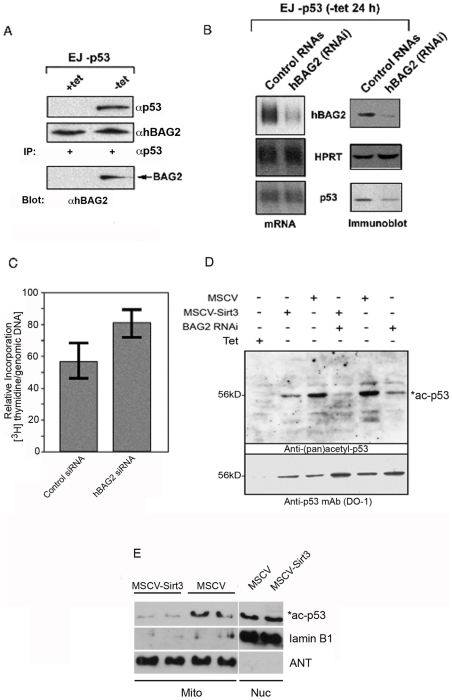
Human BAG-2 associates with p53 and stabilizes the level of p53 *in vivo*. (**A**) EJ-p53 cells were cultured to induce p53 expression upon withdrawal of tetracycline after 24 hours. As shown, input level of p53 and human BAG-2 are shown from total cell lysates obtained from the EJ-p53 cells in the presence (+tet) or withdrawal of tetracycline (-tet) from culture medium for EJ-p53 cells. Immunoblots were conducted with antisera against p53 and BAG-2 from 50 mg of total cell lysate. *Bottom*, shown is an immunoprecipitation conducted with anti -p53 followed by immunoblotting with anti –BAG-2 antibodies. (**B**) RNA interference (RNAi) of human BAG-2 was conducted with siRNAs (Ambion, sequence ID#137542) directed at endogenous human BAG-2 mRNA in the presence of p53 expression within EJ-p53 cells. Cells were maintained at 40% confluent growth in 5% CO_2_ and transfected with 7 mg of the 21nt siRNA in 30 mm Petri dishes. The control sample is representative of a transfection using scrambled 21nt RNA mixture provided by the manufacturer. In parallel cultures, both total cell RNA and protein was recovered and used to measure mRNA and protein levels by northern hybridization using cDNA probes for human BAG-2, HPRT, and p53 and immunoblotted with the antibodies corresponding to BAG-2, HPRT, and p53, respectively. (**C**) Thymidine incorporation into nascent genomic DNA was measured upon introduction of siRNA targeted against BAG-2 and scrambled RNAs in EJ-p53 cells induced for the expression of p53 after 24 hours. Values reflect the relative incorporation of [^3^H] thymidine *versus* DNA content. (**D**) Total cellular content of acetylated and total following induction of p53 in EJ-p53 cells and infection with the MSCV –hSirt3 retrovirus or transfection with small interfering RNAs (siRNA) against human BAG2 (BAG2 RNAi). Antibody against acetylated p53 as described was used to detect acetylated species of p53. (E) As shown in panel ***D***, Human embryonic lung fibroblasts IMR-90 cells were infected with MSCV-SirT3 and the MSCV control vector. Sub-cellular mitochondria and nuclear fractions were isolated and collected. Immunoblots confirming the presence of pan-acetylated p53, Lamin B1 and adenine nucleotide translocase (ANT) are shown from each sub-cellular fraction.

As a result of our studies to identify domains necessary for p53 to execute cell growth arrest in the EJ-p53 cell line (**Fig. 5**), we wanted to identify potential interacting partners for p53 through the mitchrondrial –associated senescence domain (MASD) of p53. Using a yeast two -hybrid interaction assay, we utilized the fragment of p53 between amino acids 64–209 as the bait for determining protein interactions. Our hypothesis is that protein interactions mediated through this region of p53 may influence p53's ability to direct mitochondrial –associated senescence (MAS). After screening with the yeast two-hybrid interaction assay, we had identified 14 candidates. Since our initial expectation was the possibility of re-identifying SirT3 as a candidate among those isolated clones, sequence analysis of several positive interactions resulted in the identification of one candidate protein called BCL2-associated athanogene 2 (BAG-2). Although, BAG-2 has never been shown to be associated with p53, recent studies show that BAG -related proteins compete with Hip for binding to the Hsc70/Hsp70 ATPase domain and promote protein substrate release involving both the chaperoning, folding, and degradation of wild type and mutant p53 [24], [25]. BAG-2, which is an auxiliary subunit of the carboxyl terminus of Hsp70-interacting protein complex (CHIP), functions as a co-chaperone for specific protein substrates to provide a means of interfering with proteosomal degradation [26]. In our study we identify BAG-2 as interacting with p53 in the EJ-p53 cells following induction of p53 (**Fig. 5A**). This activity seems coupled to the stability of p53 as shown (**Fig. 5B**). We show that BAG-2 influences the stability of p53 after 24 hours following tet withdrawal whereas p53 mRNA remains unaffected (**Fig. 5B**). To see if there is any abrogation of p53 function to induce growth arrest in the EJ-53 cells, thymidine incorporation analysis indicates that inhibition of BAG-2 by RNA interference (RNAi) increases thymidine incorporation as an index of cell proliferation (**Fig. 5C**). Furthermore, using an earlier time point of 18 hours following tet withdrawal we see a direct correlation between BAG-2 inhibition and the reduction in acetylated p53 species (**Fig. 5D**). This activity was further augmented by the infection with MSCV-hSirT3 retrovirus (**Fig. 5D**). Indicating that BAG-2 increases intracellular levels of acetylated p53 in direct contrast to the decrease seen following infection with SirT3 retrovirus (**Fig. 5D**). In accordance with evidence shown (**Fig. 5D**) for specific p53 deacetylation, we tested whether the SirT3 lysine deacetylase activity from human embryonic lung fibroblasts IMR-90 transduced with the MSCV-SirT3 retrovirus is specific for mitochondrial p53. Shown are the sub-cellular mitochondrial and nuclear fractions taken from cells infected MSCV-SirT3 and MSCV and assayed for pan –acetylated p53, along with protein landmarks corresponding to the nucleus and mitochondria purity. Our results demonstrate that p53 deacetylation by SirT3 is restricted to mitochondria and not the nucleus of EJ-p53 cells induced for p53 expressionafter 24 hours (**Fig. 5E**). The contribution of BAG-2 in directing some p53 activity suggest a role for BAG-2 and CHIP in maintaining native conformation possibly to stabilize p53 into a folded native condition associated with cellular stress. Despite the central role of Mdm2 in proteasomal targeting of p53, additional ubiquitin ligases were recently shown to participate in the degradation of the tumor suppressor in normal cells, including p300, Pirh2, and COP1 [24]. Although p300 seems to cooperate with Mdm2 during ubiquitylation, Pirh2 and COP1 trigger the destruction of p53 independent of Mdm2. Multiple degradation pathways apparently exist to maintain low levels of p53 in normal cells. It is unclear whether these degradation pathways are truly redundant or whether they are selectively engaged in p53 destruction dependent on cell lineage, developmental stage, or physiological situation. The complexity of p53 degradation may reflect the regulation and integration of diverse p53-mediated signaling pathways. Interestingly, we had identified mtHsp40 (Tid1), as this manuscript was being prepared, as an interacting protein with p53 (*not shown*) now confirmed to interact with cytosolic p53 under hypoxic conditions [27]. Future experiments will now take into consideration the network of different chaperones for p53 as a client protein that may monitor the quality control and salvage of p53 to enforce the activities for p53 in mitochondria.

Protein degradation in the mammalian cytoplasm and nucleus involves cooperation of the molecular chaperones Hsc70 and Hsp90 with the ubiquitin-proteasome system [50]. Of central importance to this degradation pathway is the chaperone-associated ubiquitin ligase CHIP [51]. Through binding to the carboxyl termini of Hsc70 and Hsp90, CHIP mediates the ubiquitylation of chaperone-bound client proteins in conjunction with E2 enzymes of the Ubc4/5 family and induces client degradation by the 26 S proteasome [51]. Affected chaperone clients can be broadly divided into two subclasses: (i) Hsc70- and Hsp90-associated signaling proteins, for example the glucocorticoid hormone receptor and the oncogenic receptor tyrosine kinase ErbB2 and (ii) aggregation-prone proteins that are subjected to chaperone-assisted quality control, such as misfolded cystic fibrosis transmembrane conductance regulator (CFTR) and hyperphosphorylated *tau*. However, the full range of cellular substrates of CHIP remains to be explored. Remarkably, mice that lack CHIP develop apoptosis in multiple organs after environmental challenge [52]. This seems to reflect the role of CHIP in the conformational regulation of the heat shock transcription factor but may also mirror altered associations between the chaperone machinery and diverse apoptosis regulators in the absence of CHIP [53]. As an intrinsic mediator of CHIP activity BAG-2 plays a role in regulating access of CHIP to client proteins [26]. All the BAG proteins have an approximately 45-amino acid BAG domain near the C terminus but differ markedly in their N-terminal regions. The predicted BAG-2 protein contains 211 amino acids [26]. The BAG domains of BAG-1, BAG-2, and BAG-3 interact specifically with the Hsc70 ATPase domain *in vitro* and in mammalian cells. All 3 proteins bind with high affinity to the ATPase domain of Hsc70 and inhibit its chaperone activity in a Hip-repressible manner, thereby providing a plausible mechanism for restricting p53 degradation. [24].

## Methods

### Cell culture

Use of the human bladder tumor-derived EJ-p53 cells, previously described elsewhere [2], [33], were derived from the human EJ bladder carcinoma cells, from that express a tetracycline (tet)-regulated wild-type p53 were generously provided by Dr. Stuart Aaronson (Mount Sinai School of Medicine). All cell cultures were maintained in DMEM containing 10% FBS and tetracycline or doxycycline (Sigma-Aldrich) (1 µg/ml). EJ-p53 cells were induced to express p53 by rinsing three times with PBS (without calcium and magnesium) and then replacing with tetracycline/doxycycline -depleted medium. EJ-p53 cells became senescent within 3–4 days of sustained p53 expression [2], [33]. The human IMR-90 cell line was purchased through American Type Culture Collection (ATCC) and cultured by standard cultivating techniques using media reagents and instructions provided by the supplier. Introduction of various p53 deletions by transient transfection as described previously were used to monitor p53 function [54]. An additional deletion was created to remove amino acids 64 through 209 by using PCR deletion strategy and the Quickchange mutagenesis kit (Stratagene).

### Subcellular and biochemical fractionation

Mitochondrial fractionation was achieved using the procedures essentially as described with the modifications described here [29], [55]. Sucrose step gradient was performed on EJ-p53 cells at different time points after induction of p53 by removal of tetracycline from the culture media (referred to as –tet), including 0 h (∼ +tet, control), -tet 2 h, 4 h, 6 h, 1 d, 2 d, 3 d, 4 d. The 1 ml pellet of cells was washed in 10 to 20 ml of TD buffer (134 mM NaCl, 5 mM KCl, 0.7 mM Na_2_HPO_4_, 2.5 mM Tris-HCl, pH 7.5) and centrifuged for 5 min at 2.5 krpm in a Sorvall centrifuge. Cells were resuspended in 10–12 ml of MgRS Buffer (10 mM NaCl, 1.5 mM MgCl_2_, 10 mM Tris-HCl, pH 7.5) and incubated for 10 min. Swollen cells were disrupted in a glass Dounce homogenizer to yield approximately 95% free nuclei as judged by phase contrast microscope. 2.5× MS buffer was added to a final concentration of 0.21 M mannitol, 0.07 M sucrose, 5 mM Tris-HCl,pH 7.5. Nuclei was isolated by two successive sedimentations of 5 min each at 3,000 rpm and recovered for further purity using the nuclear extraction kit (Imgenex) according to manufacturer's instructions. The supernatant was then centrifuged at 10,000 rpm for 20 min, and the pellet was resuspended in 3 ml 1× MS buffer. Loading 3 ml of 1.5 M sucrose solution on the bottom of the tube, the 3 ml of 1 M of sucrose was loaded on the interphase, and the sample was loaded on the sucrose cushion and centrifuged further at 22,000 rpm (110,000×g) at 4°C for 30 min. Mitochondria fractions were collected between the 1–1.5 interphase and sample was washed with 4 volumes of the MS buffer. Alternatively, mitochondrial fractions were recovered using the *MITOISO1®* Mitochondria Isolation Kit (Sigma) according to the manufacturer's instructions.

### Immunoblot analysis

Mitochondrial and nuclear fractions in addition to total cell lysate from each of the time points indicated and conditions used were electrophoresed using SDS-PAGE. Protein quantitation was performed using the BCA protein assay (Pierce). Between 25 – 50 µg of protein per sample was subjected to 10% SDS-PAGE and transfer to a polyvinylidene difluoride filter (PVDF) (Millipore). The filter was blocked in 10% milk/0.1% Tween 20/PBS followed by incubation with the different primary antibodies as indicated p53-specific mouse monoclonal DO-1 (Leica Microsystems); pan-acetylated p53 rabbit polyclonal antisera (lys 320, 373, 382, mixture of Millipore cat nos. 06-758, 06-915); cyclooxygenase subunit II (Cox II) (70 kD subunit, mouse monoclonal, clone AS66, Sigma-Aldrich); mtHsp70 (anti-HSPA9, Sigma-Aldrich); PCNA (mouse monoclonal, clone PC-10, Cell Signaling); 4) SirT3 (Sirtuin 3, cat no. ab75434, AbCam); Adenine dinucleotide translocase (mouse monoclonal, clone 5F51BB5AG7, MitoSciences); Lamin B1 (cat no. ab16048, AbCam). After three times washes, the blot was incubated with HRP-conjugated anti-mouse for mouse monoclonal antibodies or HRP –conjugated anti-rabbit for rabbit polyclonal antibodies, respectively (Pierce). Immunodetection was performed and then developed using the ECL system (GE Healthcare).

### *In Vitro* Protein-Protein Binding Assays

The construction of the glutathione *S*-transferase (GST)-p53 fusion was performed by standard PCR and cloning techniques constructed by cleavage of the pGEX-6P1 vector and the target DNA at appropriate restriction sites and ligation using standard recombinant DNA methodology. The GST fusion proteins were expressed in *Escherichia coli* strain BL21, and were purified and immobilized to glutathione-agarose as described previously. ^35^S-Radiolabeled full-length SirT3 was synthesized *in vitro* by use of a coupled transcription/translation system (TnT kit, Promega). The ^35^S-labeled proteins were then incubated with 50 µl of a 50% slurry of the corresponding immobilized GST fusion protein in 200–300 µl of HEMG binding buffer (40 mM HEPES, pH 7.8, 50 mM KCl, 0.2 mM EDTA, 5 mM MgCl_2_, 0.1% Triton X-100, 10% glycerol, 1.5 mM dithiothreitol, 1× Complete Protease Inhibitor (Roche Biochemical), and 0.5 mg/ml bovine serum albumin) for 1 h at 4°C with gentle rocking. The agarose beads were then washed four times with 1 ml each of HEMG buffer in the absence of protease inhibitor and bovine serum albumin. Bound proteins were eluted in 30 µl of 50 mM Tris-HCl (pH 6.8) containing 100 mM glutathione, were resolved by SDS-polyacrylamide gel electrophoresis, and were visualized and quantified by PhosphorImager analysis.

### Mitochondrial labeling with CMTMR

Chloromethyl- tetramethylrosamine methyl ester (CMTMR) is a fluorescent potentiometric dye that specifically stains mitochondria in live cells. Procedure was carried as previously described [28] and according to manufacturer's instructions (Mitotracker Orange; Molecular Probes, Invitrogen). Further immunocytochemistry with selected antibodies (*as indicated*) was performed as described previously [28] and briefly explained here. Coverslips containing paraformaldehyde treated cells were blocked with 10% goat serum at room temperature for 1 hour. Primary antibody mouse monoclonal anti-p53 antibody, 1801, 1∶1000 (Oncogene Research) was used. The coverslips were then washed 3 times with 1% NP-40 in PBS and incubated with the second antibody Cy5 goat anti-mouse Ig G (Amersham): 1∶250 at 37°C in a humidified chamber for 1 hour. Finally, coverslips were washed with 1% NP 40 in PBS three times. DNA/Nucleus staining was performed with YOYO flourescent stain (Molecular Probes, Invitrogen) according to manufacturer's instructions. The coverslips were placed in 24 well plates, and PBS was removed from each well, ice cold 100% methanol was then added and immediately removed, then 200 µl YOYO working solution (1.5 µM) was placed over fixed cell populations for 30 min at 22 C in a darkroom. The coverslips were rinsed 3 times with PBS and the coverslips were mounted onto glass microscope slides using a proper fluorescent mounting medium.

### Laser Scanning Confocal Microscopic Imaging (LSCM)

CMTMR staining, YOYO staining and immunofluroescence staining were imaged using a LSCM as previously described [28] All cells were imaged at the same levels of laser intensity, detector sensitivity, and pinhole size in order to ensure that fluorescence intensity could be compared among different coverslips. The images were saved as high resolution TIFF files and then analyzed using Northern Eclipse software (version 2.1, Empix Imaging, Inc., Mississauga, CANADA).

### Genetic screening for the rescue of p53 -induced cellular senescence

10^8^ EJ-p53 prior to tetracycline withdrawal were infected with a MaRX retroviral library (complexity 6×10^6^) prepared from mouse embryos [38]. The efficiency of infection was approximately 25%. At day 3 after infection, plates were split 1∶2 and cultured in the presence of 20 g ml^−1^ hygromycin B (Sigma, St Louis, MO). Cells were split every five days until proliferation of control cells stopped. Proliferating cells from the library-infected plates were pooled and genomic DNA was purified using the blood and cell culture DNA mini kit (Qiagen, Valencia, CA). Genomic DNA (5 mg) was treated with *Cre* recombinase, phenol extracted, ethanol precipitated, and used to transform DH10B-lac-trfA. Proviruses were recovered from zeocin-resistant bacterial colonies. From all the recovered plasmids, retroviral clone A478-34 (encoding full-length mouse *SirT3*) was one of three confirmed positive retroviruses.

### Cell culture, retroviral infection, growth curve analysis, and senescence –associated (SA) β galactosidase staining

The human SirT3 2.9 kb cDNA identified in the retroviral screen was placed in the MSCV retroviral plasmid using a bicistronic cassette with GFP (Green fluorescent protein) [56]. EJ-p53 cells were transduced with MSCV particles containing only GFP, mouse *c-myc* and human SirT3. Cumulative population doublings per passage were calculated as log_2_ (number of cells at time of subculture/number of cells plated) and plotted against total time in culture to assess replicative lifespan. Cell growth kinetics were determined by laser scanning florescence microscopy for GFP and by monitoring cell populations. [^3^H]-thymidine incorporation studies were assayed after 72 hours essentially as described [57] in EJ-p53 cells following transduction with indicated retroviruses followed by induction of p53 expression by tetracycline withdrawal for 72 hours. SA-β galactosidase activity were determined in retroviral transduced EJ-p53 with MSCV-GFP/SirT3 transduced into EJ-p53 cells prior and following induction of p53 by tetracycline withdrawal using the senescence –associated β-galactosidase Staining Kit (Cell Signaling Technology) according to manufacturer's instructions.

### Protein deacetylase assays of p53

Protein deacetylation reactions were performed essentially as previously described [58], [59]. Protein deacetylase activity was measured against a synthetic peptide corresponding to the human p53 protein sequence (HLKSKKGQSTSRHKKLMFK-C) was synthesized and purified by HPLC (Pepitogenic, Inc.). Purified synthetic peptide was radiolabeled with [^14^C] -acetylCoA (GE Healthcare) and subjected to enzymatic reactions with purified acetyltransferases CBP and PCAF *in vitro* using procedures previously described [60]. Deacetylase activity was determined from mitochondrial and nuclear fractions taken from EJ-p53 cells following 12 hours after induction of p53 expression by tetracycline withdrawal. Deacetylase activity was determined from p53, Sirt1, and Sirt3 immunopreciptates taken from nuclear and mitochondrial fractions.
